# Multidimensional scaling of diffuse gliomas: application to the 2016 World Health Organization classification system with prognostically relevant molecular subtype discovery

**DOI:** 10.1186/s40478-017-0443-7

**Published:** 2017-05-22

**Authors:** Patrick J. Cimino, Michael Zager, Lisa McFerrin, Hans-Georg Wirsching, Hamid Bolouri, Bettina Hentschel, Andreas von Deimling, David Jones, Guido Reifenberger, Michael Weller, Eric C. Holland

**Affiliations:** 1 Department of Pathology, Division of Neuropathology, University of Washington School of Medicine, 325 9th Avenue, Box 359791, Seattle, WA 98104 USA; 2 Division of Human Biology, and Seattle Tumor and Translational Research, Fred Hutchinson Cancer Research Center, 1100 Fairview Ave N., Mailstop C3-168, Seattle, WA 98109 USA; 3 Institute for Medical Informatics, Statistics and Epidemiology, University of Leipzig, Leipzig, Germany; 4 Department of Neuropathology, Heidelberg University Hospital, Heidelberg, Germany; 5 CCU Neuropathology, German Cancer Research Center (DKFZ), Heidelberg, Germany; 6 German Cancer Consortium (DKTK), Heidelberg, Germany; 7 Department of Pediatrics, Division of Pediatric Neurooncology, Heidelberg University Hospital, Heidelberg, Germany; 8 Department of Pediatric Immunology, Division of Pediatric Neurooncology, Heidelberg University Hospital, Heidelberg, Germany; 9 Department of Neuropathology, Heinrich Heine University, Duesseldorf, Germany; 10 German Cancer Consortium (DKTK), partner site Essen/Duesseldorf, German Cancer Research Center (DKFZ), Heidelberg, Germany; 11 Department of Neurology and Brain Tumor Center, University Hospital and University of Zurich, Zurich, Switzerland; 12 Department of Neurological Surgery, Alvord Brain Tumor Center, University of Washington School of Medicine, Seattle, WA USA

**Keywords:** Oncoscape, Glioma, Glioblastoma, Astrocytoma, Oligodendroglioma, Isocitrate Dehydrogenase (IDH), World Health Organization (WHO)

## Abstract

Recent updating of the World Health Organization (WHO) classification of central nervous system (CNS) tumors in 2016 demonstrates the first organized effort to restructure brain tumor classification by incorporating histomorphologic features with recurrent molecular alterations. Revised CNS tumor diagnostic criteria also attempt to reduce interobserver variability of histological interpretation and provide more accurate stratification related to clinical outcome. As an example, diffuse gliomas (WHO grades II–IV) are now molecularly stratified based upon isocitrate dehydrogenase 1 or 2 (IDH) mutational status, with gliomas of WHO grades II and III being substratified according to 1p/19q codeletion status. For now, grading of diffuse gliomas is still dependent upon histological parameters. Independent of WHO classification criteria, multidimensional scaling analysis of molecular signatures for diffuse gliomas from The Cancer Genome Atlas (TCGA) has identified distinct molecular subgroups, and allows for their visualization in 2-dimensional (2D) space. Using the web-based platform Oncoscape as a tool, we applied multidimensional scaling-derived molecular groups to the 2D visualization of the 2016 WHO classification of diffuse gliomas. Here we show that molecular multidimensional scaling of TCGA data provides 2D clustering that represents the 2016 WHO classification of diffuse gliomas. Additionally, we used this platform to successfully identify and define novel copy-number alteration-based molecular subtypes, which are independent of WHO grading, as well as predictive of clinical outcome. The prognostic utility of these molecular subtypes was further validated using an independent data set of the German Glioma Network prospective glioblastoma patient cohort.

**Electronic supplementary material:**

The online version of this article (doi:10.1186/s40478-017-0443-7) contains supplementary material, which is available to authorized users.

## Introduction

For nearly a century, classification of primary brain tumors has been based solely upon histomorphologic characteristics and presumed histogenesis of neoplastic cell types [2, 3]. Early classification systems for diffuse gliomas relied upon evaluating the histological subtype as either astrocytoma or oligodendroglioma, with further histological parameters such as nuclear atypia, mitotic figures, microvascular proliferation, and necrosis, to indicate aggressiveness, or higher-grades of gliomas [2, 20, 34, 38]. Today the most commonly used standard criteria for classifying gliomas is set forth by the World Health Organization (WHO). Originally presented in 1979, the WHO classification of central nervous system (CNS) tumors has been revised in 1993, 2000, 2007, and most recently in 2016 [23]. Prior to the 2016 classification system, WHO glioma classification was based solely upon histopathological criteria, which contains an inherent amount of interobserver variability in interpretation, leading to less predictive clinical outcomes [10, 11, 13, 21, 24, 42]. More recently, large scale genomic efforts such as those from The Genome Cancer Atlas (TCGA) have led to a considerable increase in the identification and understanding of recurrent genetic and epigenetic alterations found in diffuse gliomas, WHO grades II–IV, and have helped to define molecular and prognostic subclasses of these tumors [7–9, 30, 31, 43, 45, 46]. Such molecular alterations include mutations in the *isocitrate dehydrogenase* (*IDH*) *1* and *2* genes, codeletion of chromosome arms 1p and 19q, or hypermethylation of the gene encoding O-6-methylguanine DNA methyltransferase (*MGMT*) [4, 8, 9, 12, 22, 31, 32, 46]. To reflect the understanding of genetic and genomic contributions to glioma biology, the 2016 WHO classification introduced revised classification criteria to incorporate traditional histopathology and molecular signatures into ‘integrated’ diagnostic entities [23, 25, 26, 33]. Special attention has also been made in this new version to conceptually restructure glioma classification to consider all diffuse gliomas (astrocytomas and oligodendrogliomas) under the common header of “diffuse astrocytic and oligodendroglial tumors” [23]. Within this category, molecular alterations help to drive WHO grade II and III diagnoses, and diagnostic entities include diffuse astrocytoma designated as IDH-mutant or IDH-wildtype; anaplastic astrocytoma designated as IDH-mutant or IDH-wildtype; Oligodendroglioma, IDH-mutant, and 1p/19q-codeleted; and anaplastic oligodendroglioma, IDH-mutant, and 1p/19q-codeleted [23]. Not otherwise specified (NOS) categories of these entities are also present, but should be reserved for cases where molecular testing is not possible or where the results are not conclusive [23]. Another change in the WHO grade II and III diffuse glioma category is the discouragement of an oligoastrocytoma diagnosis [23]. In most instances, oligoastrocytoma can be refined into either the astrocytoma or oligodendroglioma category based upon molecular information [23, 39]. Glioblastoma, WHO grade IV, is now also classified according to IDH status into either glioblastoma, IDH-mutant or glioblastoma, IDH-wildtype [23]. Histological variants of glioblastoma, IDH-wildtype include gliosarcoma, giant cell glioblastoma, and epithelioid glioblastoma. Again, a NOS designation can be applied in cases of insufficient molecular information concerning the IDH mutation status.

A recent analysis of molecular signatures of TCGA diffuse glioma datasets by multidimensional scaling (MDS) showed that there are distinct groups of tumors that cluster together in 2-Dimensional (2D) space [5]. This genomic analysis incorporated data from DNA methylation, DNA copy number alterations (CNAs), and DNA single nucleotide alterations (SNAs). Major genomic factors influencing non-biased large clustering of diffuse gliomas included IDH mutational status, CpG island methylator phenotype (CIMP), polysomy of chromosome 7, monosomy of chromosome 10, and codeletion of chromosome arms 1p and 19q [5]. Regional grouping within larger clusters is also seen in association with specific genetic alterations, such as those in the genes *NRAS*, *HER2*, and *TP53*. Using this multidimensional scaling analysis, a web-based, interactive visualization platform termed Oncoscape [27] was developed for menu-driven investigation of heterogeneous clinical, pathological, and molecular parameters of various TCGA cancer datasets, including diffuse gliomas. Oncoscape, allows users to compare patients across multiple clinical and genetic features, define trait-based cohorts, align patients and cohorts along a clinical timeline, perform integrated statistical analyses, and create high-quality visualization of integrated clinical and molecular data [27]. In the present study, we leverage Oncoscape to apply 2D multidimensional scaling analysis of TCGA data to visualize relevant molecular characteristics related to the 2016 WHO classification of diffuse glioma entities. In addition, we demonstrated the utility of Oncoscape for exploring genetically defined subgroups within major diffuse glioma categories.

## Materials and methods

### Oncoscape and TCGA data visualization

TCGA point mutation and copy number data for glioblastomas as well as WHO grade II and III astrocytic and oligodendroglial tumors were downloaded from the University of California Santa Cruz cancer browser (https://genome-cancer.ucsc.edu/). Classic multidimensional scaling (MDS) of molecular data was performed as previously described [5]. The minimal TCGA tumor purity has been reported at 60-80%, which has been shown to be sufficient (>50% tumor purity) for robust detection of cancer-related copy number alterations via GISTIC 2.0 scores [35], and therefore undersampling of glioma copy number alterations is not likely to affect MDS in this study. Clinical data were obtained from the Genomic Data Commons (GDC) Data Portal from the National Institutes of Health (NIH) [16]. Data were visualized and analyzed using the interactive browser-based platform, Oncoscape (https://oncoscape.sttrcancer.org). Diffuse gliomas from the ‘gliomas (TCGA)’ dataset were visualized in 2D space with available ‘Markers and Patients’ analysis tools. To be consistent with our prior description [5], the MDS layout utilizing ‘all genes’ was used. The current online version of Oncoscape (https://oncoscape.sttrcancer.org; accessed on May 12, 2017) offers several different 2D layout options, including 13 MDS layouts and 21 principle component analysis (PCA) layouts based upon differing combinations of molecular input (copy numbers alterations, somatic nucleotide mutations, and RNAseq gene expression) and nine different gene sets. Relevant genetic and genomic alterations used for 2016 WHO diffuse glioma classification were queried in Oncoscape [23]. Also of interest for comparison, were previous 2007 histopathologic classifications and WHO grades of TCGA datasets [24]. Three main clusters were identified by MDS, and individual patients were assigned to each based upon *IDH1/2* mutational status and the presence or absence of 1p/19q codeletion (Additional file 1: Table S1).

### TCGA copy number frequency

Using GISTIC2.0 scores, copy number frequencies of TCGA gliomas were plotted using R software (Version 3.3.2, *R*Project for Statistical Computing, http://www.r-project.org/) applying the ‘copynumber’ package (http://bioconductor.org/packages/copynumber) using 0.5 and −0.5 as thresholds.

### German glioma network validation set

Glioblastoma sample molecular data (*n* = 284) from the German Glioma Network (http://www.gliomnetzwerk.de) were collected along with survival and gene methylation data as previously described [41]. Copy number alterations of individual CpG sites were evaluated based on the R package ‘conumee’ (http://bioconductor.org/packages/conumee) applying an adapted algorithm for baseline-correction. For evaluation of chromosomal segments, the median of the states of the corresponding probes was computed. Gains and losses were called using thresholds at −0.1 and 0.1 on a log2-scale as cutoff. For calling of amplifications and homozygous deletions in genes of interest thresholds at 0.6 and −0.6 were used. Segment start- and end-positions refer to reference genome GRCh37/hg19. Results are restricted to chromosomes 1, 14 and 19 as well as *CDKN2A*, *CDK4*, and *MDM2*.

### Plots and statistics

Statistical analyses were performed using R software (Version 3.3.2, *R*Project for Statistical Computing, http://www.r-project.org/). Kaplan-Meier analysis for overall survival was performed using the ‘survival’ package (https://cran.r-project.org/package=survival) with *P*-values determined by Cox proportional hazards regression. Multivariate Cox proportional hazards models including indicated variables were applied utilizing SPSS statistical software (Version 22.0, IBM). Linear regression was performed using GraphPad Prism software (Version 7.02, https://www.graphpad.com/scientific-software/prism).

## Results

### Visualizing WHO diffuse glioma classification

**Fig. 1:**
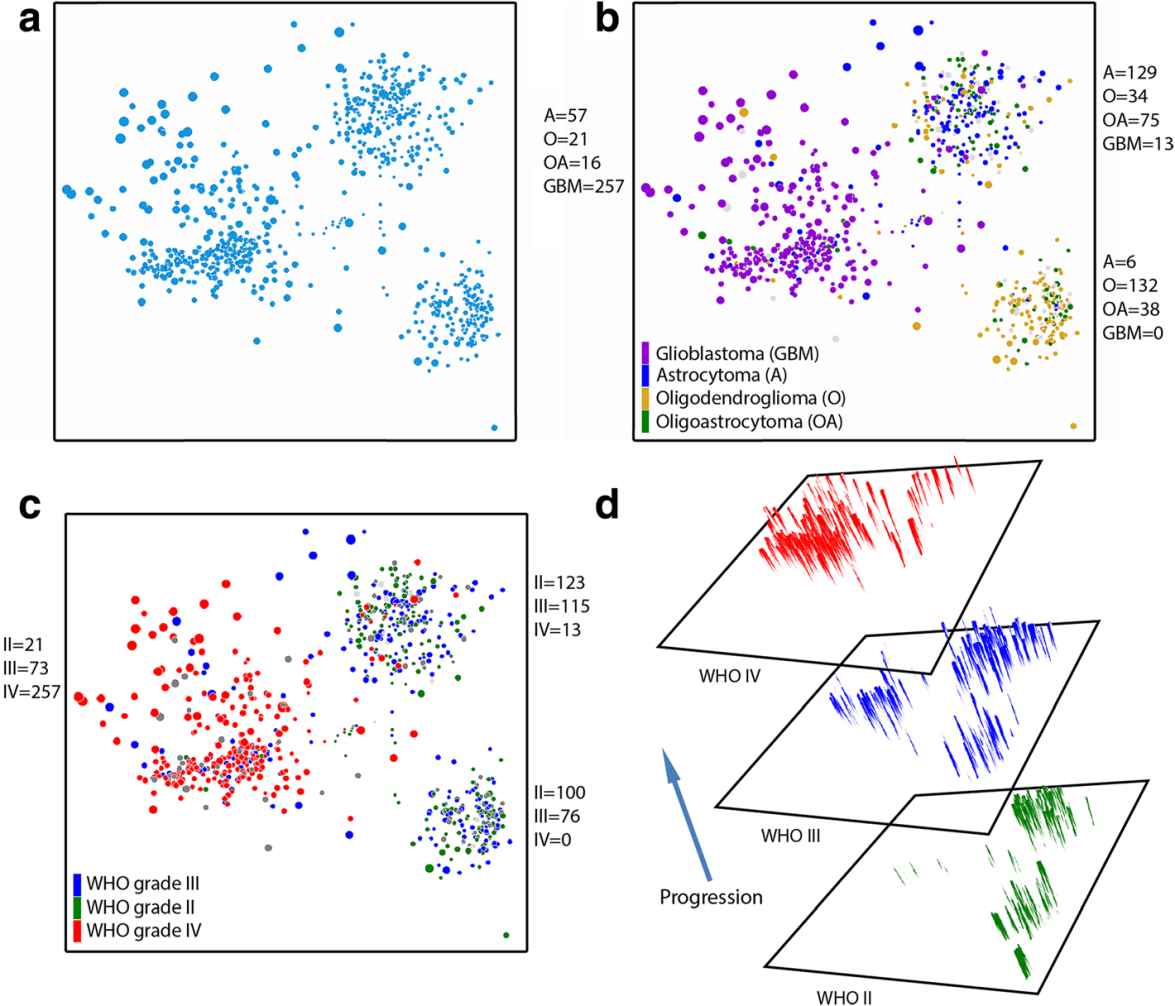
2D multidimensional scaling plots of TCGA diffuse glioma patients based on genomic data. **a** Multidimensional scaling shows that there are three main clusters. **b** 2007 WHO histopathological classification across the three main clusters (number of cases for each cluster is listed). **c** WHO grades are shown across clusters (number of cases for each cluster is listed). **d** 3D representation of WHO grading, reflecting progression of each cluster

Initially, the diffuse glioma TCGA data were visualized in relation to 2007 WHO classification criteria, including histopathology and WHO grade (Fig. 1). For each charted patient point, the dot diameter increases with increased genetic alterations (Fig. 1a). Histopathologic diagnoses as defined by the outdated 2007 WHO classification of primary brain tumors (oligodendroglioma, astrocytoma, oligoastrocytoma, glioblastoma) are not molecular cluster-specific, as each cluster contains a variable amount of histopathologic heterogeneity (Fig. 1b). This highlights the issue addressed by the current 2016 WHO classification, i.e. that histopathologic criteria alone are not entirely representative of genetic alterations in diffuse gliomas, and that the new WHO classification of integrating histopathology with molecular studies can be more reproducible for diagnostic purposes.

**Fig. 2:**
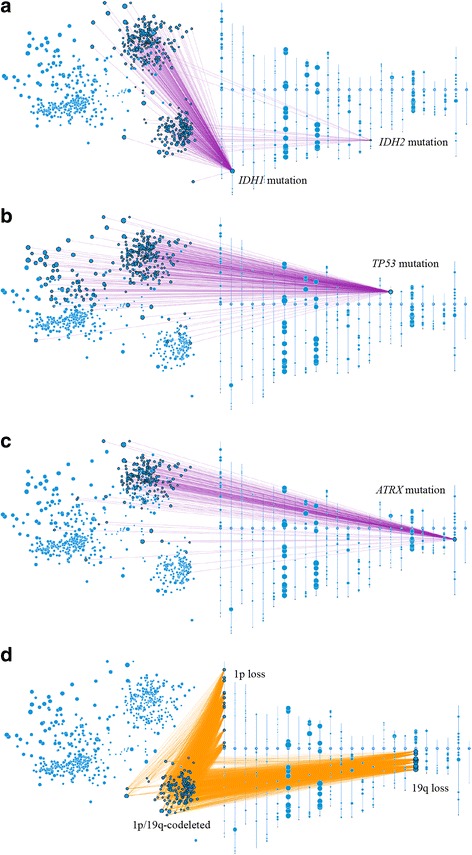
2D diffuse glioma plots with accompanying chromosomal ideograms. **a** The two main clusters on the right contain mutations in the *IDH1* and *IDH2* genes, as shown by the purple edges connecting the gene with corresponding patients. The IDH-mutant upper right cluster also carries the majority of (**b**) *TP53* and (**c**) *ATRX* gene mutations. **d** The IDH-mutant lower right cluster contains gliomas that harbor the oligodendroglioma-specific 1p/19q codeletion, as demonstrated by orange edges connecting low level copy loss chromosomal regions with corresponding affected patients. This cluster also contains a majority of the *IDH2* mutations

**Fig. 3:**
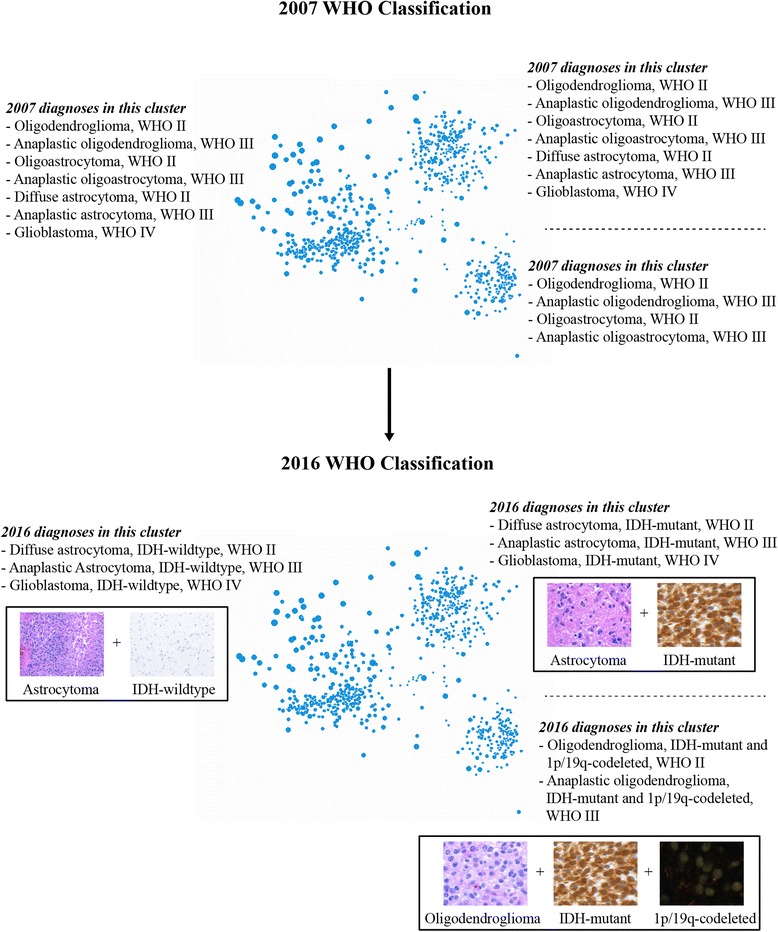
2D visualization of the revised 2016 WHO classification of diffuse gliomas. Multidimensional scaling demonstrates three major clusters of diffuse gliomas. The 2007 WHO histopathologic classifiers are heterogeneous and non-specific with regards to the three main clusters. The 2016 WHO classification aligns well with the three major clusters and can be divided into: 1) oligodendroglial tumors, IDH-mutant and 1p/19q-codeleted (WHO grades II–III); 2) astrocytic gliomas/glioblastomas, IDH-mutant (WHO grades II–IV); and 3) astrocytic gliomas/glioblastomas, IDH-wildtype (WHO grades II–IV)

Therefore, we also queried patient clusters for genetic changes corresponding to those used for the ‘integrated’ 2016 WHO classification of diffuse gliomas (Fig. 2). Presence of mutated *IDH1/2* characterizes two main clusters, and is absent from the third cluster (Fig. 2a). Mutated *IDH1* is more common and more evenly dispersed than mutated *IDH2. TP53* and *ATRX* mutations occur mostly in one of the IDH-mutant clusters (Fig. 2b,c). The other IDH-mutant cluster exclusively harbors 1p/19q codeletion (Fig. 2d). Mutations in *IDH2* are seen more frequently in the 1p/19q-codeleted cluster (Fig. 2b), and appear to have regional grouping as well, indicating a unique type of DNA structure for these types of gliomas. Consistent with prior reports [39, 40, 44], 1p chromosomal deletion was more cluster specific than 19q chromosomal deletion. WHO grades II–IV are seen in both clusters without 1p/19q codeletion, consistent with the concept that there are no WHO grade IV oligodendrogliomas [15, 28]. This observation also supports the removal of glioblastoma with oligodendroglial component as a distinct diagnostic entity [18]. Taken together, these three clusters can be designated based upon the 2016 WHO classification criteria as follows: 1) Oligodendroglial tumors, IDH-mutant, and 1p/19q-codeleted (WHO grades II–III) (*n* = 176); 2) Astrocytic gliomas/glioblastoma, IDH-mutant (WHO II–IV) (*n* = 251); and 3) Astrocytic gliomas/glioblastoma, IDH-wildtype (WHO grades II–IV) (*n* = 351) (Fig. 3).

### Cluster demographics

**Fig. 4:**
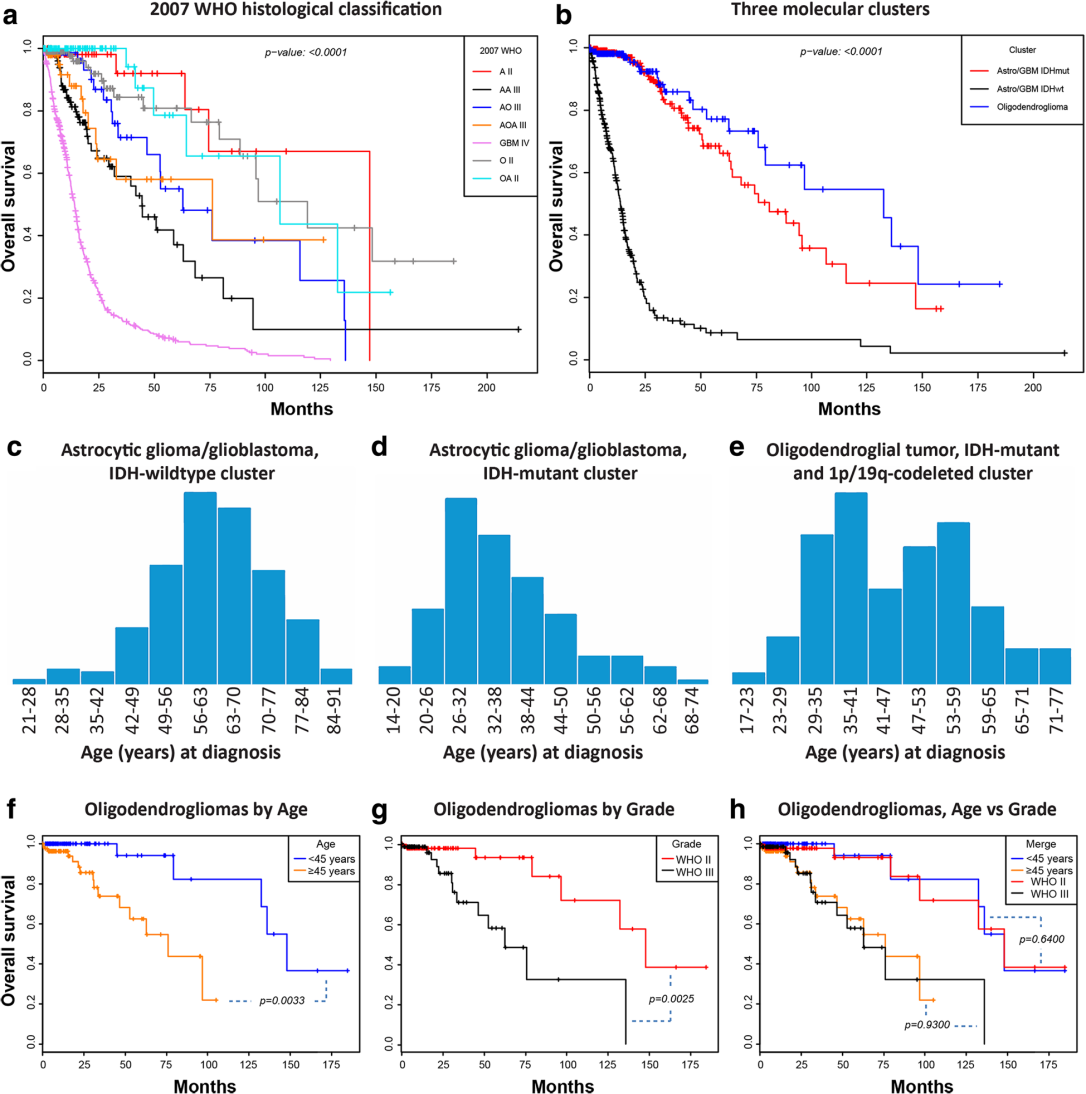
Clinical characteristics of TCGA diffuse glioma clusters. **a** Survival curves from 2007 WHO histopathological classification criteria (A = diffuse astrocytoma, O = oligodendroglioma, OA = oligoastrocytoma, AA = anaplastic astrocytoma, AO = anaplastic oligodendroglioma, AOA = anaplastic oligoastrocytoma, GBM = glioblastoma). **b** Survival comparison of three main 2D molecular clusters. **c**-**e** Age at diagnosis distribution for each cluster. Patients with astrocytic glioma/glioblastoma are older at presentation in the (**c**) IDH-wildtype cluster than in the (**d**) *IDH* mutant cluster. **e** An apparent bimodal adult age distribution is seen in the oligodendroglioma cluster, with median age of 45 years. **f** Survival of patients with tumors of the oligodendroglioma cluster stratified by age (<45 versus ≥45 years) is significantly different (*p = 0.0033*), and comparable to survival stratified by WHO grade (**g**, **h**). *P* values determined using Cox proportional hazard regression

Comparison of survival between and within the major diffuse glioma molecular clusters reflects the improved and revised 2016 WHO classification system (Fig. 4a, b) [23]. Comparison of the three molecular clusters defined by MDS demonstrates and confirms prognostic effect of IDH mutations, which is further stratified by 1p/19q codeletion status (Fig. 4b) [8, 9, 23, 26, 30–32, 40]. When looking at WHO grade IV glioblastomas, the tumors within the IDH-mutant cluster are associated with longer survival than tumors within the IDH-wildtype cluster, again consistent with prior studies of glioblastoma and the new WHO classifications [4, 17, 23, 26, 30, 46]. There are some caveats in trying to interpret survival studies from this TCGA data, especially for the WHO grade II and III diffuse gliomas, as availability of outcome data was not a main factor in selecting cases. WHO grade II and III diffuse glioma patients have longer survival than glioblastoma patients, and therefore require longer clinical follow up. In addition to survival, age at diagnosis was shown to vary between clusters (Fig. 4c–e). The astrocytic glioma/glioblastoma, IDH-wildtype cluster has the oldest age distribution peak, occurring at 56–63 years (Fig. 4c). By contrast, the astrocytic glioma/glioblastoma, IDH-mutant cluster has the youngest age distribution peak, occurring at 26–32 years (Fig. 4d). The oligodendroglial tumor cluster shows a distribution in adults with two peak ages at diagnosis at 35–41 and 53–59 year age ranges, with a median age of 45 years (Fig. 4e). The association of age within the 2016 WHO definition of IDH-mutant and 1p/19q-codeleted oligodendroglial tumors has not been described. To investigate associations of this bimodal age distribution with overall survival, the oligodendroglial tumor cluster was subdivided into two groups utilizing median age at diagnosis (45 years) as a cut-off (Fig. 4f). Kaplan-Meier survival analysis demonstrated similar survival curves when divided either by age or WHO grade (Fig. 4f-h). Additionally, hazard ratios were prognostic in a Cox regression model containing age <45 versus ≥45 (hazard ratio [HR]0.137, 95% confidence interval [CI] 0.024–0.774, *p = 0.024*), WHO grade II versus grade III (hazard ratio [HR]0.200, 95% confidence interval [CI] 0.051–0.791, *p = 0.022*) and Karnofsky performance score 90–100% versus <90% (hazard ratio [HR]0.167, 95% confidence interval [CI] 0.042–0.660, *p = 0.011*).

### Global copy number alterations of MDS clusters

**Fig. 5:**
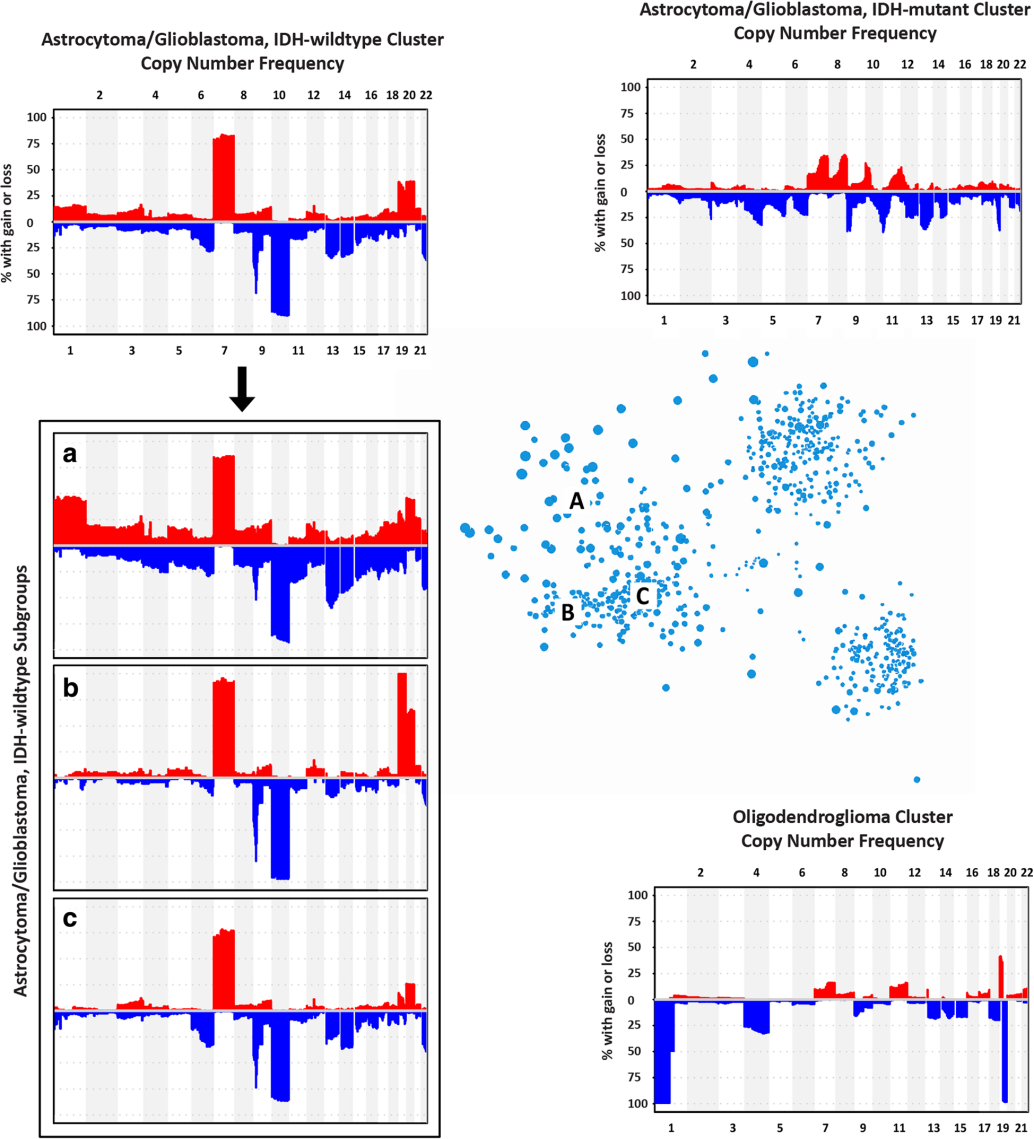
Genomic copy number alteration frequency among molecular clusters. Oligodendrogliomas are defined by 1p/19q codeletion and the second most frequent alteration is loss of chromosome 4. The IDH-mutant astrocytic glioma/glioblastoma cluster has several low level copy number alterations, including known astrocytoma-associated alterations such as 9p loss and 19q loss. IDH-wildtype diffuse gliomas have frequent polysomy chromosome 7, chromosome 10 loss, and 9p loss. The IDH-wildtype cluster can be further divided into 3 subgroups (**a**–**c**). Subgroup A is separated from B and C by either the presence of polysomy chromosome 1 or *TP53* mutations. Subgroups B and C are further separated by the presence or absence of polysomy chromosome 19

To add to the current WHO genetic classifiers of IDH mutation and 1p/19q codeletion, global copy number alteration (CNA) frequencies were analyzed across the molecular clusters defined by MDS to confirm known CNAs as well as identify new cluster-associated CNAs (Fig. 5). The oligodendroglial tumor cluster is defined by the presence of 1p/19q codeletion and the second most frequent alteration (~25%) is loss of chromosome 4. Other CNAs across oligodendrogliomas include low-level gains (chromosomes 7, 8, 11, 16, 17, 20, 21, and 22) and low-level losses (chromosomes 6, 9, 10, 12, 13, 14, 15, 18, and 22) of uncertain significance. The astrocytic glioma/glioblastoma, IDH-mutant cluster appears more heterogeneous with respect to CNA than the other clusters. It has several low- to mid-level CNAs, but unlike the other clusters, no alteration was present in >50% of the cluster. Some of the mid-level CNAs include known astrocytoma-associate alterations such as 9p loss, 10q loss, and 19q loss [6, 36]. Gain of chromosome 7 is also present in tumors of the astrocytic glioma/glioblastoma, IDH-mutant cluster, but not as frequently as in the corresponding astrocytic glioma/glioblastoma, IDH-wildtype cluster. Regardless of IDH status, astrocytic glioma/glioblastoma patients have low-frequency amplifications of 12q in the region of *CDK4* and *MDM2*. Of note, these alterations were not limited to glioblastomas. The IDH-wildtype diffuse gliomas have frequent chromosome 7 gain, chromosome 10 loss, and 9p loss, among other CNAs. Additional MDS in three dimensions (image available at http://zager.co/glioma/chart.html) demonstrates that the IDH-wildtype cluster can be divided spatially into three subgroups (designated as A–C in Fig. 5). The IDH-wildtype subgroup A is separated from B and C by chromosome 1 gain and/or *TP53* point mutations [5]. Furthermore, subgroups B and C can be simply distinguished by chromosome 19 gain. Co-gain of chromosomes 19 and 20, which has been described in a small series of long-term glioblastoma survivors [14], is frequently observed in the IDH-wildtype subgroup B. There is a significant survival difference (*p = 0.034*, Cox proportional hazards regression) between subgroups B and C, which are distinguished by gain of chromosome 19 (Additional file 1: Figure S1). Co-amplification of *CDK4* and *MDM2* further augments survival in a molecular subgroup-specific manner (Additional file 1: Figure S1).

### Identification and characterization of cluster-derived molecular subtypes

**Fig. 6:**
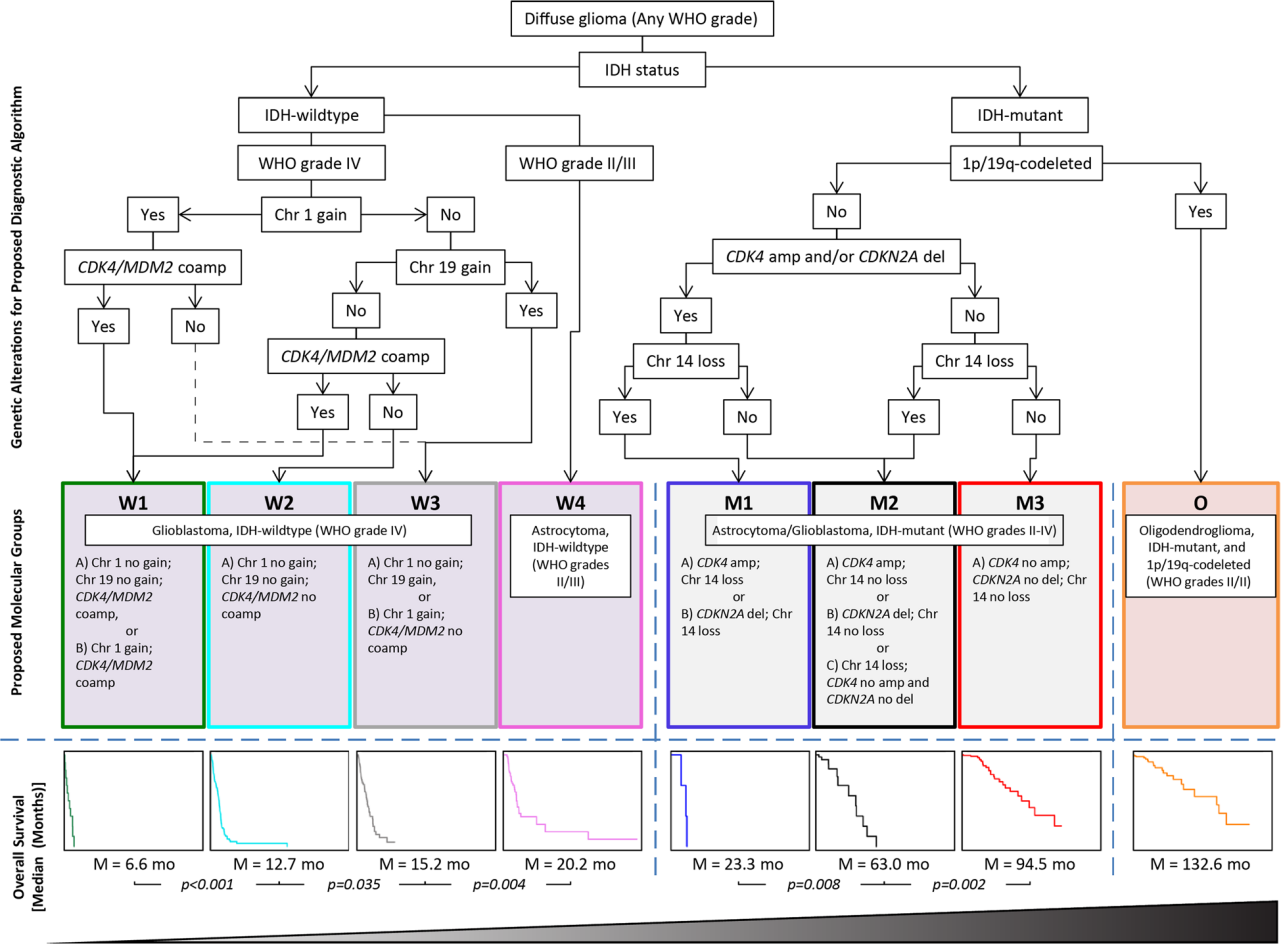
Diagnostic algorithm for 2D-mapping derived, copy number alteration-based, molecular subtypes of diffuse gliomas. Survival represent TCGA glioma dataset and P values were determined using Cox proportional hazard regression

**Fig. 7:**
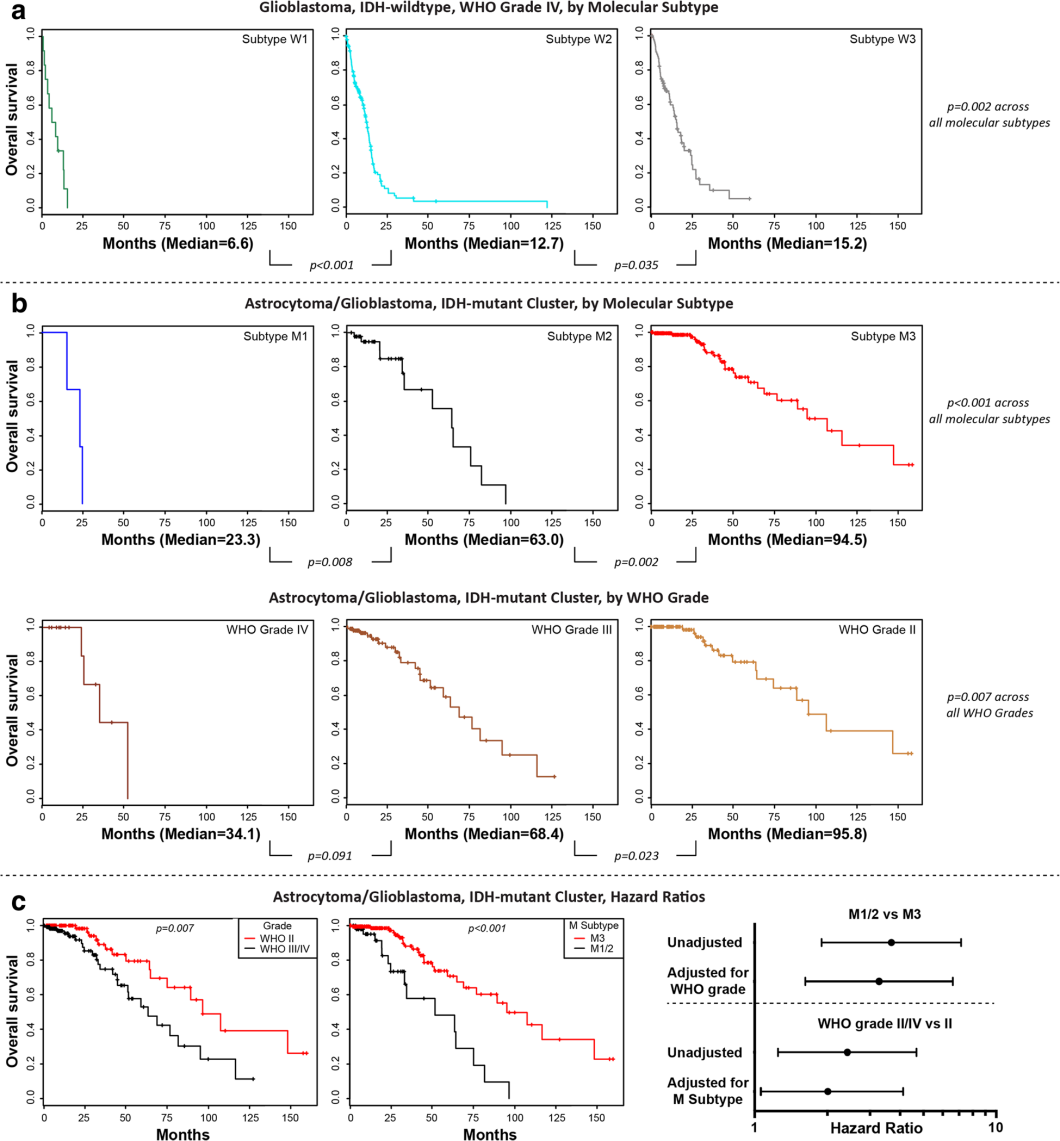
Multidimensional scale mapping derived copy number alterations forms unique prognostic molecular subtypes. **a** Glioblastoma, IDH-wildtype, WHO grade IV can be divided into three subtypes (W1–3). **b** The IDH-mutant astrocytic glioma/glioblastoma cluster can be divided into three molecular subtypes. These molecular subtypes are reflective of overall survival, and independent of WHO grade. **c** Dividing the molecular subtypes into either poor (M1/M2) or favorable (M3) groups is significantly associated with survival (Hazard ratio [HR] 3.28, 95% confidence interval [CI] 1.62–6.62, *p = 0.001*). This Hazard ratio is slightly larger, but comparable to dividing this cluster into WHO grade II versus WHO grade III/IV (HR 2.01, 95% CI 1.06–4.02, *p = 0.036*). *P* values determined using Cox proportional hazard regression

After evaluation of global CNA frequency across the astrocytic glioma/glioblastoma clusters, a small number of cluster-derived CNAs were interrogated for survival prediction and possible risk-stratification. CNA molecular subtypes are defined by chromosome 1 gain, chromosome 19 gain, and *CDK4/MDM2* co-amplification for the astrocytic glioma/glioblastoma, IDH-wildtype cluster (W1–W4), as well as *CDK4* amplification, *CDKN2A* deletion, and chromosome 14 gain for the astrocytic glioma/glioblastoma, IDH-mutant cluster (M1–M3) (Fig. 6). For the most common type of diffuse glioma, i.e. glioblastoma, IDH-wildtype, WHO Grade IV, there is a difference in overall survival across the W1–W3 molecular subtypes (*p = 0.002*, Cox proportional hazards regression, Fig. 7a), with median overall survival of 6.6 months (W1), 12.7 months (W2) and 15.2 months (W3), respectively. As these are all WHO grade IV tumors, these wildtype molecular subtypes are independent of grading. For the astrocytic glioma/glioblastoma, IDH-mutant cluster, independent of WHO grade, there is a significant overall survival difference across the M1–M3 molecular subtypes (*p < 0.001*, Cox proportional hazards regression, Fig. 7b), with median survivals of 23.3 months (M1), 63.0 months (M2) and 94.5 months (M3). Segregation by WHO grade was prognostic within the astrocytic glioma/glioblastoma, IDH-mutant cluster, yielding a median overall survival of 34.1 months (WHO Grade IV), 68.4 months (WHO Grade III) and 95.8 months (WHO Grade II), respectively (*p = 0.007*, Cox proportional hazards regression, Fig. 7b). In patients within the astrocytic glioma/glioblastoma, IDH-mutant glioma cluster, median overall survival with WHO grade III/IV versus WHO grade II was 63.0 versus 95.8 months (*p = 0.007*, Cox proportional hazards regression, Fig. 7c). Segregation of these patients by M1/2 versus M3 molecular subgroups yielded similar survival proportions of 51.2 versus 94.5 months (*p < 0.001*, Cox proportional hazards regression, Fig. 7c). The association with overall survival was retained for M1/2 versus M3 upon adjustment for WHO grade (Hazard ratio [HR] 3.28, 95% confidence interval [CI] 1.62–6.62, *p = 0.001*), and vice versa for WHO grade III/IV versus grade II upon adjustment for molecular subgroup (HR 2.01, 95% CI 1.06–4.02, *p = 0.036*, Fig. 7c). Survival curves within the astrocytic glioma/glioblastoma, IDH-mutant cluster based on molecular subtypes (M1-3) or WHO grade (II–IV) are somewhat comparable, with a slightly stronger association with overall survival for molecular subtyping. However, molecular classification may be more reliable than grading between pathologists as the exact criteria for defining a WHO grade II diffuse astrocytoma versus WHO grade III anaplastic astrocytoma are not well-defined for resection material and may be associated with interobserver variability [1, 23, 26, 29, 42].

### Validation of cluster-derived molecular subtypes

**Fig. 8:**
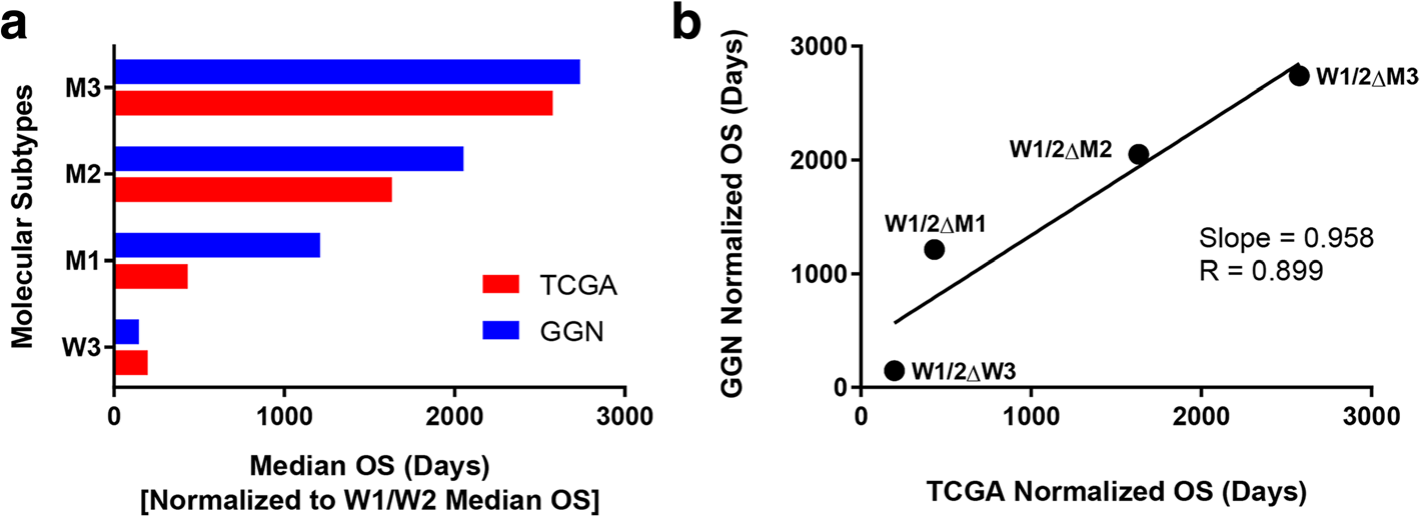
Prognostic validation of The Cancer Genome Atlas (TCGA) cluster-derived molecular subtypes in a large cohort from the German Glioma Network (GGN). **a** Bar graph showing normalized median overall survival (OS) compared to baseline with similar trends for TCGA and GGN datasets. **b** Linear regression analysis demonstrating equivalent ratio of normalized molecular subtype OS between TCGA and GGN data sets

To test the potential clinical utility of cluster-derived molecular subtypes, an independent large validation data set (*n* = 284) of glioblastomas from the German Glioma Network (GGN) was evaluated. Comparing WHO grade IV glioblastomas, median overall survival of the GGN cohort (18.9 months) versus the TCGA cohort (13.5 months) was longer (*p < 0.001*, Cox proportional hazards regression). A caveat to this comparison is that the TCGA gliomas are WHO grade II–IV while the GGN gliomas are all WHO grade IV, which should not be a significant confounder as the molecular subtypes appear to be grade independent (Fig. 7) and in addition, the GGN data set has longer overall survival. Overall survival data in the GGN cohort were normalized for validation to account for the difference in median survival between the GGN and TCGA cohorts. In the GGN cohort the W1 and W2 subtypes did not show difference (*p = 0.913*, Cox proportional hazards regression) in survival so their combined median overall survival was used for baseline normalization. Normalized median overall survival for each molecular subtype showed similar trends for TCGA and GGN data sets (Fig. 8a). Additionally, linear regression shows a nearly 1:1 overall ratio (slope = 0.958, *R* = 0.899) comparing TCGA and GGN overall survival by molecular subtypes (Fig. 8b). In summary, overall survival times of the cluster-derived molecular subtypes identified in the TCGA discovery set were comparable in the GGN validation set.

## Discussion

Visualization of adult diffuse gliomas by multidimensional analysis represented in Oncoscape provides convenient graphical representation of the revised 2016 WHO classification system in 2D space. Three main Oncoscape clusters represent the new risk-stratification classification by IDH mutation and 1p/19q codeletion status. Subgroups based upon additional molecular alterations within these clusters are emerging, but are still yet to be completely defined [5]. The molecular groups are not well-reflected by the 2007 WHO histopathological criteria alone. In fact, each molecular cluster shows variable heterogeneity of histopathological subtypes of diffuse gliomas. This observation reinforces the concept of interobserver variability of diagnoses based upon histology alone, and highlights why integrating molecular alterations in diffuse gliomas increases diagnostic accuracy.

In addition to illustrating the inherent interobserver variability of histopathologic only classification of diffuse gliomas, Oncoscape also reflects why the diagnosis of (mixed) oligoastrocytoma is discouraged in the new WHO system and how ‘oligoastrocytoma’ easily resolves into either oligodendroglioma or astrocytoma entities [13, 23, 33, 37]. If oligoastrocytoma truly existed as a specific biological entity, these cases would be expected to exist between the IDH-mutant oligodendroglial and astrocytic glioma clusters shown in Fig. 1b, however, no such cases are seen. Furthermore, histopathological ‘oligoastrocytoma’ cases are predominantly evenly distributed and completely embedded within the oligodendroglial tumor and astrocytic glioma/glioblastoma, IDH-mutant clusters, further arguing against oligoastrocytoma as a distinct entity. However, we do note that there are reported rare cases of true oligoastrocytomas with distinct regions of either molecular oligodendroglioma or astrocytoma features [19], and the WHO does allow for designation of these gliomas as oligoastrocytoma, NOS [23]. However, where such rare molecular biphenotypic cases fall into place on the Oncoscape map has yet to be determined.

A current area for future clarification and refinement in the WHO classification system is that of grading [26]. While molecular alterations were incorporated into the 2016 WHO classification system, grading of diffuse gliomas did not change from the prior 2007 edition [23, 24, 26]. It appears that molecular alterations are strong drivers of clinical behavior, and may be considered as a first stratifier, as IDH-mutant diffuse gliomas clinically behave better than IDH-wildtype diffuse gliomas across all grades [4, 8, 17, 23, 26, 31, 32]. For example, determination of IDH mutational or ‘Oncoscape’ cluster status, may be considered as baseline diagnostic criteria. After the baseline diagnosis is established, cluster-specific grading may be warranted, either histologically or molecularly. On the histologic side, there is some existing literature that supports this type of molecular stratification first, followed by grading. Using a specific mitotic index independent of WHO grading, mitotic counting has been shown to stratify IDH-wildtype, but not IDH-mutant astrocytomas [31]. This suggests that there may be yet to be determined cluster-specific mitotic indices for future WHO grading of diffuse gliomas that better predict clinical outcome. On the molecular side, we present data in this study supporting prognostic heterogeneity within major diffuse glioma clusters, which in some aspects is identified by conventional grading, but is even better identified by an additional set of molecular markers. These results provide evidence of the utility for ‘molecular grading’ within major subgroups of diffuse gliomas.

Along with reflecting changes in WHO classification of diffuse gliomas, some patterns of genetic alterations become readily apparent by this visualization tool, as was the case for the bimodal age distribution of the IDH-mutant, and 1p/19q-codeleted Oligodendroglioma cluster (Fig. 4) and cluster-specific gene amplifications (Fig. 5). For example, the size of dots, representing the amount of genetic alterations in a single sample as shown in Fig. 1, demonstrates the amount of genetic variation within clusters. The IDH-mutant, and 1p/19q-codeleted oligodendroglial tumor cluster is enriched for the smallest points, indicating a genomically stable group, while the astrocytic glioma/glioblastoma, IDH-wildtype cluster has the largest points, representing more frequent mutations and copy number aberrations in this more aggressive type of glioma. The group with an intermediate clinical outcome, the astrocytic glioma/glioblastoma, IDH-mutant cluster, has the most variability in dot size, indicating a genomically more heterogeneous group. Perhaps in diffuse gliomas, the IDH mutational pathway of oncogenesis leads to some genomic stability with 1p/19q codeletion further potentiating relative genomic stability.

With the TCGA dataset, there are current limitations with regards to placement of some diffuse glioma histologic subtypes on the 2D Oncoscape map. For example, it is not clear where gemistocytic astrocytoma, IDH-mutant (a histological variant of diffuse astrocytoma, IDH-mutant) [23], would be distributed in the astrocytic glioma/glioblastoma, IDH-mutant cluster, or if regional clustering would occur at all. In addition, there are glioblastoma, IDH-wildtype histological variants that have an unknown cluster distribution, either because of lack of annotation or lack of sequencing. These glioblastoma variants include giant cell glioblastoma, gliosarcoma, and epithelioid glioblastoma [23]. They are presumed to exist in the IDH-wildtype cluster, but any specific regionality or grouping is unknown. Gliosarcoma and giant cell glioblastoma tend to lack *EGFR* amplification, and are therefore likely not associated with *EGFR*-amplified cases in the IDH-wildtype cluster. Epithelioid glioblastomas harbor the BRAF-V600E mutation in about half of all cases [23]. There are eleven cases of diffuse gliomas with *BRAF* single nucleotide alterations present exclusively in the IDH-wildtype cluster (not shown), however, they are of various grades (2 WHO grade II; 2 WHO grade III; 4 WHO grade IV; 2 not graded) and appear not to group together. Therefore, it will take increased numbers of properly annotated glioblastoma histological subtypes to resolve their spatial distribution, if any. Along these lines, the current datasets for brain tumors in Oncoscape are limited to the diffuse gliomas from the TCGA. Additional efforts are necessary to characterize other CNS tumor types (other glial, ependymal, glioneuronal, pineal, embryonal, meningeal, etc.) by multidimensional scaling analysis in order to compare molecular genetic structures and make associations between and amongst histologically disparate brain tumor types.

## Conclusions

The ability to visualize brain tumor datasets in 2D space relative to pathologic diagnosis and molecular alterations with tools such as Oncoscape, affords the possibility of using such a tool as a reference point for clinical utility. On the clinical side, molecular information derived from a specific patient’s surgical material can be queried in the reference dataset, and survival and treatment strategies of similar patients can inform prognosis and risk-stratification in real-time. This may have further utility in interdisciplinary settings such as neuro-oncology tumor boards, where management decisions are discussed and planned. This has the potential to ease the decision making process and contribute to the application of precision medicine for individual patients. Given the time and financial constraints that can be associated with whole exome/genome 2D mapping for an individual patient, a more limited or targeted analysis may be more prudent. In this setting, cluster-derived molecular subtypes as the ones described in this study (W1–4, M1–3), may be a more appropriate way to risk-stratify in a time-sensitive and cost-effective manner. Beyond brain tumors, Oncoscape can be used to visualize and analyze additional cancer datasets for diagnostic and translational purposes. All 33 TCGA datasets encompassing several organ systems are available for analysis in Oncoscape. Perhaps 2D molecular analysis of this type can help visualize distinct clusters of various other cancers, and help to push the solid tumors from these organ systems to move into integrated diagnoses and risk-stratification, similar to that of neoplasms of the central nervous system.

## Additional file

Additional file 1: Supplementary Material. (PDF 190 kb)
