# Supplementary material for: Multidimensional scaling of diffuse gliomas: application to the 2016 World Health Organization classification system with prognostically relevant molecular subtype discovery
Source: Acta Neuropathol Commun. 2017 May 22;5:39. doi: 10.1186/s40478-017-0443-7 (PMC5439117; doi:10.1186/s40478-017-0443-7)
Supplement: Supplementary file 1 — Supplementary Material. (PDF 190 kb) [file 40478_2017_443_MOESM1_ESM.pdf]

## Supplementary Data

**Supplementary Fig. 1** Glioblastoma (GBM), *IDH* wildtype, WHO grade IV subgroups have differential outcomes with respect to *CDK4/MDM2* co-amplification. (A) The WHO grade IV GBMs show a statistically significant survival difference between subgroup B and subgroup C (*p=0.034*), which on a copy number level, can be distinguished by chromosome 19 gain. (B) The presence of *CDK4/MDM2* co-amplification in subgroup A shows a trend for poor survival. (C) Subgroup B does not show statistically significant or trending survival differences with *CDK4/MDM2* co-amplification. (D) Subgroup C does show a significant (*p=0.033*) decrease in survival in the presence of *CDK4/MDM2* co-amplification. P values determined using Cox proportional hazard regression.

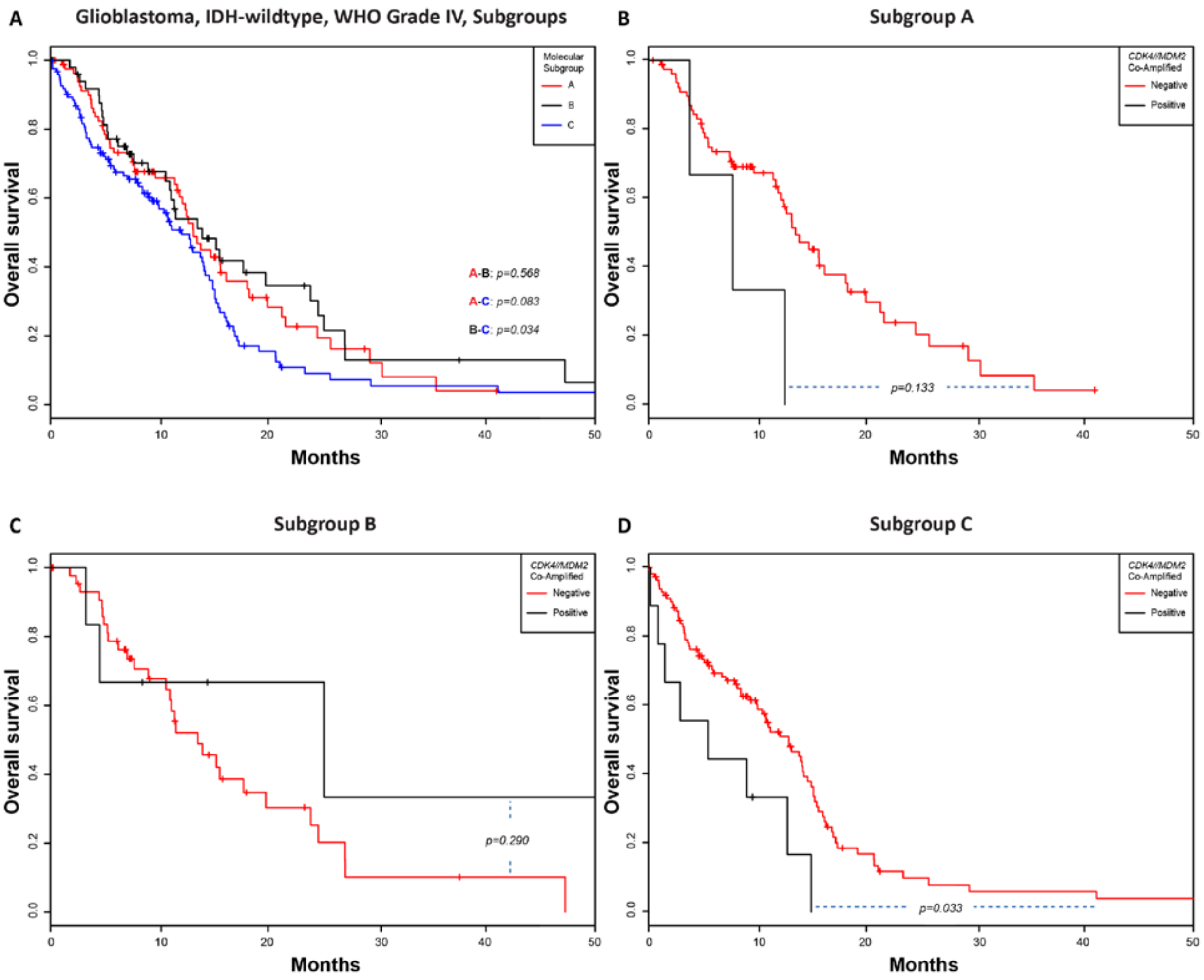

**Supplementary Table 1**

| **Cluster** | **Astrocytoma/GBM, *IDH* mutant** | **Astrocytoma/GBM, *IDH* wildtype** | **Oligodendroglioma, *IDH* mutant, 1p/19q codeleted** |
|---|---|---|---|
| **TCGA Identifier** | TCGA-02-2483-01, | TCGA-02-0003-01, | TCGA-CS-5390-01, |
| | TCGA-06-0128-01, | TCGA-02-0033-01, | TCGA-CS-5394-01, |
| | TCGA-06-0129-01, | TCGA-02-0047-01, | TCGA-CS-5396-01, |
| | TCGA-06-2570-01, | TCGA-02-0055-01, | TCGA-CS-6668-01, |
| | TCGA-06-6389-01, | TCGA-02-2470-01, | TCGA-CS-6670-01, |
| | TCGA-06-6701-01, | TCGA-02-2485-01, | TCGA-DB-5274-01, |
| | TCGA-14-1456-01, | TCGA-02-2486-01, | TCGA-DB-5278-01, |
| | TCGA-14-4157-01, | TCGA-06-0119-01, | TCGA-DB-5279-01, |
| | TCGA-15-1444-01, | TCGA-06-0122-01, | TCGA-DB-A4XA-01, |
| | TCGA-19-2629-01, | TCGA-06-0124-01, | TCGA-DB-A4XG-01, |
| | TCGA-26-1442-01, | TCGA-06-0126-01, | TCGA-DB-A4XH-01, |
| | TCGA-27-2521-01, | TCGA-06-0130-01, | TCGA-DB-A64L-01, |
| | TCGA-32-4208-01, | TCGA-06-0132-01, | TCGA-DB-A64P-01, |
| | TCGA-CS-4938-01, | TCGA-06-0137-01, | TCGA-DB-A64Q-01, |
| | TCGA-CS-4942-01, | TCGA-06-0139-01, | TCGA-DB-A64R-01, |
| | TCGA-CS-4943-01, | TCGA-06-0140-01, | TCGA-DB-A64U-01, |
| | TCGA-CS-4944-01, | TCGA-06-0141-01, | TCGA-DB-A64V-01, |
| | TCGA-CS-5393-01, | TCGA-06-0142-01, | TCGA-DB-A64W-01, |
| | TCGA-CS-6290-01, | TCGA-06-0145-01, | TCGA-DB-A75K-01, |
| | TCGA-CS-6665-01, | TCGA-06-0151-01, | TCGA-DH-5141-01, |
| | TCGA-CS-6666-01, | TCGA-06-0152-01, | TCGA-DH-5144-01, |
| | TCGA-CS-6667-01, | TCGA-06-0154-01, | TCGA-DH-A669-01, |
| | TCGA-DB-5270-01, | TCGA-06-0155-01, | TCGA-DH-A66F-01, |
| | TCGA-DB-5273-01, | TCGA-06-0157-01, | TCGA-DH-A7UR-01, |
| | TCGA-DB-5275-01, | TCGA-06-0158-01, | TCGA-DH-A7US-01, |
| | TCGA-DB-5276-01, | TCGA-06-0165-01, | TCGA-DU-5849-01, |
| | TCGA-DB-5277-01, | TCGA-06-0166-01, | TCGA-DU-5870-01, |
| | TCGA-DB-5280-01, | TCGA-06-0168-01, | TCGA-DU-5874-01, |
| | TCGA-DB-5281-01, | TCGA-06-0169-01, | TCGA-DU-6393-01, |
| | TCGA-DB-A4X9-01, | TCGA-06-0173-01, | TCGA-DU-6394-01, |
| | TCGA-DB-A4XB-01, | TCGA-06-0174-01, | TCGA-DU-6397-01, |
| | TCGA-DB-A4XC-01, | TCGA-06-0178-01, | TCGA-DU-6400-01, |
| | TCGA-DB-A4XD-01, | TCGA-06-0184-01, | TCGA-DU-6410-01, |
| | TCGA-DB-A4XE-01, | TCGA-06-0185-01, | TCGA-DU-7009-01, |
| | TCGA-DB-A4XF-01, | TCGA-06-0188-01, | TCGA-DU-7018-01, |
| | TCGA-DB-A64S-01, | TCGA-06-0189-01, | TCGA-DU-7294-01, |
| | TCGA-DB-A64X-01, | TCGA-06-0192-01, | TCGA-DU-7300-01, |
| | TCGA-DB-A75L-01, | TCGA-06-0195-01, | TCGA-DU-7302-01, |
| | TCGA-DB-A75M-01, | TCGA-06-0209-01, | TCGA-DU-8164-01, |
| | TCGA-DB-A75O-01, | TCGA-06-0213-01, | TCGA-DU-8168-01, |
| | TCGA-DH-5142-01, | TCGA-06-0214-01, | TCGA-DU-A6S2-01, |
| | TCGA-DH-5143-01, | TCGA-06-0216-01, | TCGA-DU-A6S3-01, |
| | TCGA-DH-A66B-01, | TCGA-06-0219-01, | TCGA-DU-A6S6-01, |

| | | | |
|---|---|---|---|
| | TCGA-DH-A66D-01,<br>TCGA-DH-A66G-01,<br>TCGA-DH-A7UT-01,<br>TCGA-DH-A7UU-01,<br>TCGA-DH-A7UV-01,<br>TCGA-DU-5851-01,<br>TCGA-DU-5853-01,<br>TCGA-DU-5855-01,<br>TCGA-DU-5871-01,<br>TCGA-DU-5872-01,<br>TCGA-DU-6395-01,<br>TCGA-DU-6396-01,<br>TCGA-DU-6401-01,<br>TCGA-DU-6407-01,<br>TCGA-DU-6408-01,<br>TCGA-DU-6542-01,<br>TCGA-DU-7007-01,<br>TCGA-DU-7008-01,<br>TCGA-DU-7010-01,<br>TCGA-DU-7011-01,<br>TCGA-DU-7015-01,<br>TCGA-DU-7019-01,<br>TCGA-DU-7298-01,<br>TCGA-DU-7299-01,<br>TCGA-DU-7301-01,<br>TCGA-DU-7304-01,<br>TCGA-DU-7306-01,<br>TCGA-DU-7309-01,<br>TCGA-DU-8163-01,<br>TCGA-DU-8166-01,<br>TCGA-DU-8167-01,<br>TCGA-DU-A5TP-01,<br>TCGA-DU-A5TR-01,<br>TCGA-DU-A5TS-01,<br>TCGA-DU-A5TU-01,<br>TCGA-DU-A5TW-01,<br>TCGA-DU-A6S7-01,<br>TCGA-DU-A76O-01,<br>TCGA-DU-A7T8-01,<br>TCGA-DU-A7TA-01,<br>TCGA-DU-A7TC-01,<br>TCGA-E1-5302-01,<br>TCGA-E1-5303-01,<br>TCGA-E1-5304-01,<br>TCGA-E1-5305-01,<br>TCGA-E1-5307-01,<br>TCGA-E1-A7YE-01,<br>TCGA-E1-A7YH-01, | TCGA-06-0237-01,<br>TCGA-06-0238-01,<br>TCGA-06-0240-01,<br>TCGA-06-0241-01,<br>TCGA-06-0644-01,<br>TCGA-06-0645-01,<br>TCGA-06-0646-01,<br>TCGA-06-0648-01,<br>TCGA-06-0649-01,<br>TCGA-06-0650-01,<br>TCGA-06-0686-01,<br>TCGA-06-0743-01,<br>TCGA-06-0744-01,<br>TCGA-06-0745-01,<br>TCGA-06-0747-01,<br>TCGA-06-0749-01,<br>TCGA-06-0750-01,<br>TCGA-06-0875-01,<br>TCGA-06-0876-01,<br>TCGA-06-0877-01,<br>TCGA-06-0878-01,<br>TCGA-06-0879-01,<br>TCGA-06-0881-01,<br>TCGA-06-0882-01,<br>TCGA-06-0939-01,<br>TCGA-06-1804-01,<br>TCGA-06-1806-01,<br>TCGA-06-2557-01,<br>TCGA-06-2558-01,<br>TCGA-06-2559-01,<br>TCGA-06-2561-01,<br>TCGA-06-2562-01,<br>TCGA-06-2563-01,<br>TCGA-06-2564-01,<br>TCGA-06-2565-01,<br>TCGA-06-2567-01,<br>TCGA-06-2569-01,<br>TCGA-06-5408-01,<br>TCGA-06-5410-01,<br>TCGA-06-5411-01,<br>TCGA-06-5412-01,<br>TCGA-06-5413-01,<br>TCGA-06-5414-01,<br>TCGA-06-5415-01,<br>TCGA-06-5418-01,<br>TCGA-06-5856-01,<br>TCGA-06-5858-01,<br>TCGA-06-5859-01, | TCGA-DU-A6S8-01,<br>TCGA-DU-A76R-01,<br>TCGA-DU-A7T6-01,<br>TCGA-DU-A7TG-01,<br>TCGA-E1-5311-01,<br>TCGA-E1-5318-01,<br>TCGA-E1-5319-01,<br>TCGA-E1-5322-01,<br>TCGA-E1-A7YO-01,<br>TCGA-E1-A7YS-01,<br>TCGA-E1-A7YY-01,<br>TCGA-EZ-7264-01,<br>TCGA-F6-A8O3-01,<br>TCGA-FG-5962-01,<br>TCGA-FG-5964-01,<br>TCGA-FG-7634-01,<br>TCGA-FG-7638-01,<br>TCGA-FG-7641-01,<br>TCGA-FG-8186-01,<br>TCGA-FG-8187-01,<br>TCGA-FG-8189-01,<br>TCGA-FG-A60K-01,<br>TCGA-FG-A6IZ-01,<br>TCGA-FG-A6J1-01,<br>TCGA-FG-A710-01,<br>TCGA-FG-A713-01,<br>TCGA-HT-7467-01,<br>TCGA-HT-7468-01,<br>TCGA-HT-7471-01,<br>TCGA-HT-7480-01,<br>TCGA-HT-7481-01,<br>TCGA-HT-7605-01,<br>TCGA-HT-7607-01,<br>TCGA-HT-7608-01,<br>TCGA-HT-7616-01,<br>TCGA-HT-7620-01,<br>TCGA-HT-7677-01,<br>TCGA-HT-7681-01,<br>TCGA-HT-7687-01,<br>TCGA-HT-7692-01,<br>TCGA-HT-7694-01,<br>TCGA-HT-7695-01,<br>TCGA-HT-7856-01,<br>TCGA-HT-7874-01,<br>TCGA-HT-7875-01,<br>TCGA-HT-7877-01,<br>TCGA-HT-7881-01,<br>TCGA-HT-8010-01, |

| | | | |
|---|---|---|---|
| | TCGA-E1-A7YI-01,<br>TCGA-E1-A7YK-01,<br>TCGA-E1-A7YU-01,<br>TCGA-E1-A7YV-01,<br>TCGA-E1-A7YW-01,<br>TCGA-E1-A7Z3-01,<br>TCGA-E1-A7Z4-01,<br>TCGA-E1-A7Z6-01,<br>TCGA-F6-A8O4-01,<br>TCGA-FG-5965-01,<br>TCGA-FG-6689-01,<br>TCGA-FG-6690-01,<br>TCGA-FG-6691-01,<br>TCGA-FG-7636-01,<br>TCGA-FG-8182-01,<br>TCGA-FG-8185-01,<br>TCGA-FG-8188-01,<br>TCGA-FG-8191-01,<br>TCGA-FG-A4MT-01,<br>TCGA-FG-A4MX-01,<br>TCGA-FG-A4MY-01,<br>TCGA-FG-A60J-01,<br>TCGA-FG-A60L-01,<br>TCGA-FG-A6J3-01,<br>TCGA-FG-A70Y-01,<br>TCGA-FG-A711-01,<br>TCGA-FG-A87N-01,<br>TCGA-FN-7833-01,<br>TCGA-HT-7470-01,<br>TCGA-HT-7472-01,<br>TCGA-HT-7473-01,<br>TCGA-HT-7474-01,<br>TCGA-HT-7475-01,<br>TCGA-HT-7476-01,<br>TCGA-HT-7477-01,<br>TCGA-HT-7478-01,<br>TCGA-HT-7479-01,<br>TCGA-HT-7482-01,<br>TCGA-HT-7483-01,<br>TCGA-HT-7485-01,<br>TCGA-HT-7601-01,<br>TCGA-HT-7602-01,<br>TCGA-HT-7603-01,<br>TCGA-HT-7604-01,<br>TCGA-HT-7606-01,<br>TCGA-HT-7609-01,<br>TCGA-HT-7610-01,<br>TCGA-HT-7611-01, | TCGA-06-6388-01,<br>TCGA-06-6390-01,<br>TCGA-06-6391-01,<br>TCGA-06-6693-01,<br>TCGA-06-6694-01,<br>TCGA-06-6695-01,<br>TCGA-06-6697-01,<br>TCGA-06-6698-01,<br>TCGA-06-6699-01,<br>TCGA-06-6700-01,<br>TCGA-08-0386-01,<br>TCGA-12-0615-01,<br>TCGA-12-0616-01,<br>TCGA-12-0618-01,<br>TCGA-12-0619-01,<br>TCGA-12-0688-01,<br>TCGA-12-0692-01,<br>TCGA-12-0821-01,<br>TCGA-12-3649-01,<br>TCGA-12-3650-01,<br>TCGA-12-3651-01,<br>TCGA-12-3653-01,<br>TCGA-12-5295-01,<br>TCGA-12-5299-01,<br>TCGA-12-5301-01,<br>TCGA-14-0740-01,<br>TCGA-14-0781-01,<br>TCGA-14-0786-01,<br>TCGA-14-0787-01,<br>TCGA-14-0789-01,<br>TCGA-14-0790-01,<br>TCGA-14-0813-01,<br>TCGA-14-0817-01,<br>TCGA-14-0862-01,<br>TCGA-14-0871-01,<br>TCGA-14-1034-01,<br>TCGA-14-1395-01,<br>TCGA-14-1450-01,<br>TCGA-14-1823-01,<br>TCGA-14-1825-01,<br>TCGA-14-1829-01,<br>TCGA-14-2554-01,<br>TCGA-15-0742-01,<br>TCGA-16-0846-01,<br>TCGA-16-0861-01,<br>TCGA-16-1045-01,<br>TCGA-19-1390-01,<br>TCGA-19-2619-01, | TCGA-HT-8012-01,<br>TCGA-HT-8105-01,<br>TCGA-HT-8109-01,<br>TCGA-HT-8113-01,<br>TCGA-HT-A4DV-01,<br>TCGA-HT-A5R9-01,<br>TCGA-HT-A615-01,<br>TCGA-HT-A619-01,<br>TCGA-HT-A74K-01,<br>TCGA-HT-A74L-01,<br>TCGA-HW-7486-01,<br>TCGA-HW-7487-01,<br>TCGA-HW-7491-01,<br>TCGA-HW-8322-01,<br>TCGA-HW-A5KJ-01,<br>TCGA-IK-8125-01,<br>TCGA-KT-A74X-01,<br>TCGA-P5-A5ET-01,<br>TCGA-P5-A5EX-01,<br>TCGA-P5-A5F0-01,<br>TCGA-P5-A72Z-01,<br>TCGA-P5-A730-01,<br>TCGA-P5-A737-01,<br>TCGA-P5-A77W-01,<br>TCGA-P5-A77X-01,<br>TCGA-P5-A781-01,<br>TCGA-QH-A65R-01,<br>TCGA-QH-A65V-01,<br>TCGA-QH-A65X-01,<br>TCGA-QH-A65Z-01,<br>TCGA-QH-A6CU-01,<br>TCGA-QH-A6CY-01,<br>TCGA-QH-A6CZ-01,<br>TCGA-QH-A6X4-01,<br>TCGA-QH-A6X5-01,<br>TCGA-QH-A6X8-01,<br>TCGA-QH-A86X-01,<br>TCGA-R8-A6MK-01,<br>TCGA-R8-A6ML-01,<br>TCGA-R8-A6MO-01,<br>TCGA-R8-A73M-01,<br>TCGA-RY-A83X-01,<br>TCGA-RY-A83Y-01,<br>TCGA-RY-A840-01,<br>TCGA-RY-A847-01,<br>TCGA-S9-A6TW-01,<br>TCGA-S9-A6TX-01,<br>TCGA-S9-A6TY-01, |

| | | | |
|---|---|---|---|
| | TCGA-HT-7676-01,<br>TCGA-HT-7684-01,<br>TCGA-HT-7686-01,<br>TCGA-HT-7688-01,<br>TCGA-HT-7689-01,<br>TCGA-HT-7690-01,<br>TCGA-HT-7693-01,<br>TCGA-HT-7855-01,<br>TCGA-HT-7858-01,<br>TCGA-HT-7873-01,<br>TCGA-HT-7879-01,<br>TCGA-HT-7880-01,<br>TCGA-HT-7884-01,<br>TCGA-HT-7902-01,<br>TCGA-HT-8013-01,<br>TCGA-HT-8018-01,<br>TCGA-HT-8106-01,<br>TCGA-HT-8108-01,<br>TCGA-HT-8111-01,<br>TCGA-HT-8114-01,<br>TCGA-HT-8563-01,<br>TCGA-HT-A5R5-01,<br>TCGA-HT-A5R7-01,<br>TCGA-HT-A5RB-01,<br>TCGA-HT-A614-01,<br>TCGA-HT-A616-01,<br>TCGA-HT-A618-01,<br>TCGA-HT-A61B-01,<br>TCGA-HT-A74J-01,<br>TCGA-HT-A74O-01,<br>TCGA-HW-7489-01,<br>TCGA-HW-7490-01,<br>TCGA-HW-8319-01,<br>TCGA-HW-8320-01,<br>TCGA-HW-8321-01,<br>TCGA-HW-A5KL-01,<br>TCGA-HW-A5KM-01,<br>TCGA-IK-7675-01,<br>TCGA-P5-A5EU-01,<br>TCGA-P5-A5EV-01,<br>TCGA-P5-A5EW-01,<br>TCGA-P5-A5EZ-01,<br>TCGA-P5-A5F1-01,<br>TCGA-P5-A5F2-01,<br>TCGA-P5-A5F4-01,<br>TCGA-P5-A72W-01,<br>TCGA-P5-A72X-01,<br>TCGA-P5-A731-01, | TCGA-19-2620-01,<br>TCGA-19-2623-01,<br>TCGA-19-2624-01,<br>TCGA-19-2625-01,<br>TCGA-19-2631-01,<br>TCGA-19-5947-01,<br>TCGA-19-5950-01,<br>TCGA-19-5951-01,<br>TCGA-19-5952-01,<br>TCGA-19-5953-01,<br>TCGA-19-5954-01,<br>TCGA-19-5955-01,<br>TCGA-19-5958-01,<br>TCGA-19-5959-01,<br>TCGA-19-5960-01,<br>TCGA-26-1439-01,<br>TCGA-26-5132-01,<br>TCGA-26-5133-01,<br>TCGA-26-5134-01,<br>TCGA-26-5135-01,<br>TCGA-26-5136-01,<br>TCGA-26-5139-01,<br>TCGA-26-6173-01,<br>TCGA-26-6174-01,<br>TCGA-27-1830-01,<br>TCGA-27-1831-01,<br>TCGA-27-1832-01,<br>TCGA-27-1833-01,<br>TCGA-27-1834-01,<br>TCGA-27-1835-01,<br>TCGA-27-1836-01,<br>TCGA-27-1837-01,<br>TCGA-27-1838-01,<br>TCGA-27-2518-01,<br>TCGA-27-2519-01,<br>TCGA-27-2523-01,<br>TCGA-27-2524-01,<br>TCGA-27-2526-01,<br>TCGA-27-2527-01,<br>TCGA-27-2528-01,<br>TCGA-28-1747-01,<br>TCGA-28-1753-01,<br>TCGA-28-2502-01,<br>TCGA-28-2509-01,<br>TCGA-28-2513-01,<br>TCGA-28-2514-01,<br>TCGA-28-5204-01,<br>TCGA-28-5207-01, | TCGA-S9-A6U2-01,<br>TCGA-S9-A6U5-01,<br>TCGA-S9-A6UB-01,<br>TCGA-S9-A6WD-01,<br>TCGA-S9-A6WE-01,<br>TCGA-S9-A6WH-01,<br>TCGA-S9-A6WI-01,<br>TCGA-S9-A6WL-01,<br>TCGA-S9-A6WN-01,<br>TCGA-S9-A6WP-01,<br>TCGA-S9-A7IQ-01,<br>TCGA-S9-A7IY-01,<br>TCGA-S9-A7J1-01,<br>TCGA-S9-A7J2-01,<br>TCGA-S9-A7J3-01,<br>TCGA-S9-A7QY-01,<br>TCGA-S9-A7QZ-01,<br>TCGA-S9-A7R1-01,<br>TCGA-TM-A7C5-01,<br>TCGA-TM-A84G-01,<br>TCGA-TM-A84M-01,<br>TCGA-TM-A84O-01,<br>TCGA-TM-A84R-01,<br>TCGA-TM-A84S-01,<br>TCGA-TQ-A7RG-01,<br>TCGA-TQ-A7RI-01,<br>TCGA-TQ-A7RN-01,<br>TCGA-TQ-A7RO-01,<br>TCGA-TQ-A7RQ-01,<br>TCGA-TQ-A7RS-01,<br>TCGA-TQ-A7RU-01,<br>TCGA-VM-A8CA-01,<br>TCGA-VM-A8CB-01,<br>TCGA-VM-A8CE-01,<br>TCGA-VV-A829-01,<br>TCGA-VW-A7QS-01,<br>TCGA-W9-A837-01 |

| | | | |
|---|---|---|---|
| | TCGA-P5-A733-01,<br>TCGA-P5-A735-01,<br>TCGA-P5-A736-01,<br>TCGA-P5-A780-01,<br>TCGA-QH-A65S-01,<br>TCGA-QH-A6CW-01,<br>TCGA-QH-A6X3-01,<br>TCGA-QH-A6X9-01,<br>TCGA-QH-A6XA-01,<br>TCGA-QH-A870-01,<br>TCGA-RY-A83Z-01,<br>TCGA-RY-A843-01,<br>TCGA-RY-A845-01,<br>TCGA-S9-A6TS-01,<br>TCGA-S9-A6TU-01,<br>TCGA-S9-A6TV-01,<br>TCGA-S9-A6TZ-01,<br>TCGA-S9-A6U1-01,<br>TCGA-S9-A6U6-01,<br>TCGA-S9-A6U8-01,<br>TCGA-S9-A6U9-01,<br>TCGA-S9-A6WG-01,<br>TCGA-S9-A6WO-01,<br>TCGA-S9-A6WQ-01,<br>TCGA-S9-A7IS-01,<br>TCGA-S9-A7IZ-01,<br>TCGA-S9-A7J0-01,<br>TCGA-S9-A7QW-01,<br>TCGA-S9-A7QX-01,<br>TCGA-S9-A7R3-01,<br>TCGA-S9-A7R4-01,<br>TCGA-S9-A7R7-01,<br>TCGA-S9-A7R8-01,<br>TCGA-S9-A89Z-01,<br>TCGA-TM-A7C4-01,<br>TCGA-TM-A7CA-01,<br>TCGA-TM-A7CF-01,<br>TCGA-TM-A84F-01,<br>TCGA-TM-A84H-01,<br>TCGA-TM-A84I-01,<br>TCGA-TM-A84L-01,<br>TCGA-TM-A84Q-01,<br>TCGA-TM-A84T-01,<br>TCGA-TQ-A7RF-01,<br>TCGA-TQ-A7RH-01,<br>TCGA-TQ-A7RJ-01,<br>TCGA-TQ-A7RK-01,<br>TCGA-TQ-A7RM-01, | TCGA-28-5208-01,<br>TCGA-28-5209-01,<br>TCGA-28-5211-01,<br>TCGA-28-5213-01,<br>TCGA-28-5214-01,<br>TCGA-28-5215-01,<br>TCGA-28-5216-01,<br>TCGA-28-5218-01,<br>TCGA-28-5219-01,<br>TCGA-28-5220-01,<br>TCGA-28-6450-01,<br>TCGA-32-1970-01,<br>TCGA-32-1977-01,<br>TCGA-32-1979-01,<br>TCGA-32-1980-01,<br>TCGA-32-1982-01,<br>TCGA-32-1986-01,<br>TCGA-3 2-1991-01,<br>TCGA-32-2491-01,<br>TCGA-32-2494-01,<br>TCGA-32-2495-01,<br>TCGA-32-2615-01,<br>TCGA-32-2632-01,<br>TCGA-32-2634-01,<br>TCGA-32-2638-01,<br>TCGA-32-4210-01,<br>TCGA-32-4211-01,<br>TCGA-32-4213-01,<br>TCGA-32-4719-01,<br>TCGA-32-5222-01,<br>TCGA-41-2571-01,<br>TCGA-41-2572-01,<br>TCGA-41-2573-01,<br>TCGA-41-2575-01,<br>TCGA-41-3392-01,<br>TCGA-41-3393-01,<br>TCGA-41-3915-01,<br>TCGA-41-4097-01,<br>TCGA-41-5651-01,<br>TCGA-41-6646-01,<br>TCGA-74-6573-01,<br>TCGA-74-6575-01,<br>TCGA-74-6577-01,<br>TCGA-74-6578-01,<br>TCGA-74-6584-01,<br>TCGA-76-4925-01,<br>TCGA-76-4926-01,<br>TCGA-76-4928-01, | |

| | | | |
|---|---|---|---|
| | TCGA-TQ-A7RR-01,<br>TCGA-TQ-A7RV-01,<br>TCGA-TQ-A7RW-01,<br>TCGA-TQ-A8XE-01,<br>TCGA-VM-A8C8-01,<br>TCGA-VM-A8CF-01,<br>TCGA-VM-A8CH-01,<br>TCGA-VV-A86M-01,<br>TCGA-WH-A86K-01,<br>TCGA-WY-A858-01,<br>TCGA-WY-A859-01,<br>TCGA-WY-A85A-01,<br>TCGA-WY-A85B-01,<br>TCGA-WY-A85C-01,<br>TCGA-WY-A85D-01,<br>TCGA-WY-A85E-01 | TCGA-76-4929-01,<br>TCGA-76-4931-01,<br>TCGA-76-4934-01,<br>TCGA-76-4935-01,<br>TCGA-76-6191-01,<br>TCGA-76-6192-01,<br>TCGA-76-6193-01,<br>TCGA-76-6280-01,<br>TCGA-76-6282-01,<br>TCGA-76-6283-01,<br>TCGA-76-6285-01,<br>TCGA-76-6286-01,<br>TCGA-76-6656-01,<br>TCGA-76-6657-01,<br>TCGA-76-6660-01,<br>TCGA-76-6661-01,<br>TCGA-76-6662-01,<br>TCGA-76-6663-01,<br>TCGA-76-6664-01,<br>TCGA-81-5910-01,<br>TCGA-81-5911-01,<br>TCGA-87-5896-01,<br>TCGA-CS-4941-01,<br>TCGA-CS-5397-01,<br>TCGA-CS-6188-01,<br>TCGA-DB-A75P-01,<br>TCGA-DU-5847-01,<br>TCGA-DU-5854-01,<br>TCGA-DU-6392-01,<br>TCGA-DU-6402-01,<br>TCGA-DU-6405-01,<br>TCGA-DU-7006-01,<br>TCGA-DU-7012-01,<br>TCGA-DU-7013-01,<br>TCGA-DU-7290-01,<br>TCGA-DU-7292-01,<br>TCGA-DU-8158-01,<br>TCGA-DU-A5TY-01,<br>TCGA-DU-A7TJ-01,<br>TCGA-E1-A7YD-01,<br>TCGA-E1-A7YJ-01,<br>TCGA-E1-A7YL-01,<br>TCGA-E1-A7YM-01,<br>TCGA-E1-A7YN-01,<br>TCGA-FG-5963-01,<br>TCGA-FG-6688-01,<br>TCGA-FG-A87Q-01,<br>TCGA-HT-7680-01, | |

| | | | |
|---|---|---|---|
| | | TCGA-HT-7691-01,<br>TCGA-HT-7854-01,<br>TCGA-HT-7857-01,<br>TCGA-HT-7860-01,<br>TCGA-HT-8011-01,<br>TCGA-HT-8015-01,<br>TCGA-HT-8104-01,<br>TCGA-HT-8110-01,<br>TCGA-HT-8564-01,<br>TCGA-HT-A4DS-01,<br>TCGA-HT-A5RA-01,<br>TCGA-HT-A5RC-01,<br>TCGA-HT-A74H-01,<br>TCGA-HW-A5KK-01,<br>TCGA-KT-A7W1-01,<br>TCGA-P5-A5EY-01,<br>TCGA-QH-A6CS-01,<br>TCGA-QH-A6CX-01,<br>TCGA-QH-A6XC-01,<br>TCGA-S9-A6U0-01,<br>TCGA-S9-A6UA-01,<br>TCGA-S9-A6WM-01,<br>TCGA-S9-A7IX-01,<br>TCGA-S9-A7R2-01,<br>TCGA-S9-A89V-01,<br>TCGA-TM-A7C3-01,<br>TCGA-TM-A84B-01,<br>TCGA-TM-A84C-01,<br>TCGA-VM-A8C9-01,<br>TCGA-VM-A8CD-01,<br>TCGA-VW-A8FI-01 | |
